# Inhibition of MLKL Attenuates Necroptotic Cell Death in a Murine Cell Model of Hepatic Ischaemia Injury

**DOI:** 10.3390/jcm10020212

**Published:** 2021-01-08

**Authors:** Raji Baidya, Jérémie Gautheron, Darrell H. G. Crawford, Haolu Wang, Kim R. Bridle

**Affiliations:** 1 Faculty of Medicine, The University of Queensland, Brisbane, Queensland 40006, Australia; r.baidya@uqconnect.edu.au (R.B.); d.crawford@uq.edu.au (D.H.G.C.); 2 Gallipoli Medical Research Institute, Brisbane, Queensland 4120, Australia; h.wang21@uq.edu.au; 3 Centre de Recherche Saint-Antoine (CRSA), Inserm, Sorbonne University, 75012 Paris, France; jeremie.gautheron@inserm.fr; 4 Institute of Cardiometabolism and Nutrition (ICAN), 75013 Paris, France; 5 Diamantina Institute, The University of Queensland, Brisbane, Queensland 4102, Australia

**Keywords:** ischaemia-reperfusion injury, necroptosis, liver transplantation, steatosis, apoptosis, in vitro

## Abstract

Background: Steatosis in donor livers poses a major risk of organ dysfunction due to their susceptibility to ischaemia-reperfusion (I/R) injury during transplant. Necroptosis, a novel form of programmed cell death, is orchestrated by receptor-interacting protein kinase 1 (RIPK1), receptor-interacting protein kinase 3 (RIPK3) and mixed-lineage kinase domain-like pseudokinase (MLKL), has been implicated in I/R injury. Here we investigated the mechanisms of cell death pathways in an in vitro model of hepato-steatotic ischaemia. Methods: Free fatty acid (FFA) treated alpha mouse liver 12 (AML-12) cells were incubated in oxygen-glucose-deprivation (OGD) conditions as seen during ischaemia. Results: We found that OGD triggered upregulation of insoluble fraction of RIPK3 and MLKL in FFA + OGD cells compared to FFA control cells. We report that intervention with small interfering (si) MLKL and siRIPK3 significantly attenuated cell death in FFA + OGD cells. Absence of activated CASPASE8 and cleaved-CASPASE3, no change in the expression of CASPASE1 and prostaglandin-endoperoxide synthase 2 (*Ptgs2)* in FFA + OGD treated cells compared to FFA control cells indicated that apoptosis, pyroptosis and ferroptosis, respectively, are unlikely to be active in this model. Conclusion: Our findings indicate that RIPK3-MLKL dependent necroptosis contributed to cell death in our in vitro model. Both MLKL and RIPK3 are promising therapeutic targets to inhibit necroptosis during ischaemic injury in fatty liver.

## 1. Introduction

Liver transplantation is the only remaining treatment for patients with end stage liver disease. With the increasing rate of obesity, the global prevalence of hepatic steatosis is estimated to be about 10–24% [1]. Increasing rates of chronic liver disease have resulted in an increased number of patients requiring liver transplantation. However, a shortage of available donor livers is a restricting factor in liver transplantation programs. This increasing discrepancy between demand and the donor pool of organs has forced the utilization of liver grafts from marginal donors that are also known as extended-criteria donors (ECD) [2,3,4]. An ECD liver is defined as an organ with an increased risk of primary non function (PNF) or delayed graft failure that causes an increased risk of morbidity or mortality in the recipient [5]. With the emergence of non-alcoholic fatty liver disease (NAFLD), hepatic steatosis is quite common in organ donors and studies have estimated the presence of steatosis in liver procurements of 9–26% [6,7,8] and at least 5–10% of donated livers are rejected for transplant due to steatosis [9,10]. These discarded livers could represent a large pool of ECD livers which are essential to meet the increasing demand for liver transplantation.

Ischaemic injury is regarded as a common event in liver transplantation. Previous studies have reported that steatotic liver has reduced tolerance and increased vulnerability to ischaemia-reperfusion (I/R) injury and is associated with PNF, delayed graft dysfunction, postsurgical biliary complications and increased morbidity [6,11,12,13]. However, the underlying mechanism contributing to steatotic I/R injury remains controversial. The potential use of fatty livers will remain restricted if the mystery surrounding the mechanism is unresolved. Studies from the past decade have indicated the key role of necroptosis during pathogenesis of liver diseases which includes hepatic I/R injury [14,15,16]. Necroptosis is a recently described form of programmed cell death which shares a common pathway of apoptosis but is morphologically similar to necrosis. Many studies have demonstrated that necroptosis plays a fundamental role during liver I/R injury [16,17].

Necroptotic molecules, receptor-interacting protein kinase 1 (RIPK1) and receptor-interacting protein kinase 3 (RIPK3) are known to act as executors of necroptosis and mixed-lineage kinase domain-like pseudokinase (MLKL) works as an effector during necroptotic cell death. Recent studies have demonstrated the critical involvement of RIPK3 and MLKL in I/R injury in various organ systems as well as in liver injury. RIPK3 participates in protein-protein interaction through the RIP homotypic interact motif (RHIM) with RIPK1 and the individual phosphorylation of RIPK1, RIPK3 and MLKL results in formation of the necrosome [18,19]. MLKL then functions down-stream of RIPK3 and is the final stage protein in the necroptotic signalling cascade [20,21]. Upon activation, MLKL migrates to the plasma membrane causing its disintegration and as a consequence cellular contents are released, resulting in necroptotic cell death [22,23].

In this study, we developed an in vitro model of hepatic steatosis undergoing ischaemic injury by using alpha mouse liver 12 (AML-12) cell line and explored the cell death pathways underlying ischaemic insults after oxygen glucose deprivation (OGD) treatment. We demonstrated that cells underwent RIPK3-MLKL dependent necroptotic cell death upon exposure to OGD. Our results demonstrated no significant changes in the expression level of proteins associated with apoptosis, pyroptosis nor ferroptosis, suggesting that necroptosis is the major cell death pathway involved in our model. In addition, MLKL small interfering RNA (siRNA) was able to reduce the necroptotic cell death triggered post OGD. Targeting MLKL may be a promising therapeutic approach to inhibit necroptosis during I/R injury in steatotic liver, with the ultimate goal of increasing the donor pool for liver transplantation.

## 2. Materials and Methods

### 2.1. Cell Culture

AML-12 cells were obtained from ATCC (CRl-2254, Manassas, VA, USA) and cultured with Dulbecco’s modified eagle medium/Nutrient mixture F-12 (DMEM/F12) (Cat #11320-033, Gibco, Life Technologies Corporation, New York, NY, USA) containing 10% foetal bovine serum (Cat# 10099-141, Gibco, Life Technologies Corporation, New York, NY, USA), 10 µg/mL insulin, 5.5 µg/mL transferrin, 5 ng/mL selenium (Cat# 51300-044, Life Technologies Corporation, New York, NY, USA) and 40 ng/mL dexamethasone. CRISPR/Cas9-edited Ripk3-deficient AML-12 were a kind gift from J. Gautheron (Sorbonne University, Inserm, Centre de Recherche Saint-Antoine, Paris, France). Cells were maintained at 37 °C, 5% CO_2_ humidified incubator and passaged at 70% confluence.

### 2.2. Free Fatty Acid Treatment

Mycoplasma free cells were seeded at a density of 8 × 10^4^ cells per well in 12-well plate and incubated for 24 h. For the free fatty acid (FFA) treatment, a mixture of sodium salt of oleate and palmitate (Cat# 07501 and P9767 respectively, Sigma-Aldrich, St Louis, MO, USA) was prepared in a ratio of 2:1. Briefly, stock solution of 80 mM sodium oleate and 40 mM sodium palmitate were prepared in 0.01M sodium hydroxide solution and dissolved at 70 °C. Then, a 12 mM stock FFA solution was prepared containing a 2:1 ratio of sodium oleate and sodium palmitate in 8% bovine serum albumin (BSA) (Cat#A8806-5G, Sigma-Aldrich, St Louis, MO, USA) and maintained at 37 °C to keep the fatty acids in solution. The solution was immediately mixed in prewarmed culture media to make a final concentration of 2 mM and cells were cultured for a further 24 h in FFA media.

### 2.3. Oxygen Glucose Deprivation (OGD) Treatment

After 24 h of FFA treatment, cells were prepared for OGD treatment to mimic ischaemia as described by other investigators [23]. Briefly, FFA loaded cells were washed twice with serum free glucose free DMEM media (Cat# 11966-05, Gibco, Life Technologies Corporation, New York, NY, USA) and transferred to the same media and placed in a hypoxic incubator with the supply of 1% O_2_, 94% N_2_, 5% CO_2_ for 12 h at 37 °C to induce ischaemia prior to collection for further tests.

### 2.4. Drug Administration

GSK’872 (Cat# S8465, Selleck, Houston, TX, USA) was dissolved in dimethyl sulfoxide (DMSO) and diluted in culture media. Cells were pre-treated with 50 µm GSK’872 during FFA treatment for 24 h before OGD. zVAD-FMK (Cat# S7023, Selleck, Houston, TX, USA) was dissolved in DMSO and diluted in culture media. Dose dependent analysis for zVAD-FMK effect was performed at the concentration of 1, 10, 50 and 100 µm. Control cells were treated with DMSO.

### 2.5. Cell Viability Assay

Cell viability of different groups of treated cells was determined by Cell Titre-Blue^®^ Cell Viability Assay (Cat# G8081, Promega, Madison, WI, USA). Briefly, 1 × 10^4^ cells/well were seeded in 96-well plates followed by FFA treatment and OGD treatment. 20 µL of cell viability reagent was added to each well of a 96 well plate and was incubated at 37 °C for 1 h. The fluorescence intensity was measured at 560Ex/590Em in a plate reader (Infinite F200, Tecan, Maennedorf, Switzerland). Cell viability was performed in triplicate and a no cell control was used to account for background fluorescence.

### 2.6. Oil Red-O Sstaining and Quantification

Intracellular fat infiltration in AML-12 cells was determined by Oil Red-O staining. Oil Red- O is a lysochrome diazo dye and it stains neutral triglycerides and lipids. Briefly, a stock solution of 0.3% oil Red -O (Cat# C150, ProSciTech, Thuringowa Central, Australia) was prepared in 99% isopropanol solution overnight. FFA treated cells were washed twice with 1× PBS. Precautions were taken to not to disrupt hepatocyte monolayers. The cells were fixed in 10% formalin solution for 1 h. A working solution was prepared by combining Oil Red-O stock solution and deionized water in a 3:2 ratio. The formalin fixed cells were gently rinsed with deionised water twice and then incubated in 60% isopropanol for 3 min. Isopropanol was removed and cells were incubated in oil red-O working solution for 5 min. Finally, cells were washed with tap water and left to dry and observed under an inverted microscope. The intracellular fat accumulation was quantified following treatment with 200 µL extracting reagent, DMSO. Extracted DMSO was placed in 96 well plates and absorbance was measured at 540 nM. DMSO alone was used as negative control.

### 2.7. Quantitative Real-Time PCR

Ribonucleic acid (RNA) was isolated from AML-12 cells using Trizol according to manufacturer’s guidelines (Cat# 15596018, Ambion, Life Technologies Corporation, New York, NY, USA). cDNA was synthesized by following the manufacturer’s instructions (Cat# BIO-65054, Bioline, Memphis, TN, USA). Quantitative real-time PCR (qPCR) reactions were performed using the ViiA 7 real-time PCR machine (Applied Bio-system, Carlsbad, CA, USA). Ribosomal protein L27 (*Rpl27*), retention in endoplasmic reticulum sorting receptor 1 (*RER1*) and 18S ribosomal RNA (*18S rRNA*) were used as housekeeping genes. Negative controls included no-template and non-transcribed RNA samples for each primer set. All samples were run in duplicate for each gene. qPCR was performed for solute carrier family 2, facilitated glucose transporter member 1 (*Slc2a1*), vascular endothelial growth factor (*Vegf*) and prostaglandin-endoperoxide synthase 2 (*Ptgs2)*. The primer sequences are as shown in Table 1.

### 2.8. Western Blot Assay

For total protein isolation, AML-12 cells were lysed using radio-immunoprecipitation assay (RIPA) buffer (Cat # 89900, Thermo Fisher Scientific, Rockford, IL, USA) and cOmplete mini, EDTA-free Protease Inhibitor (Cat# 11836170001, Roche Diagnostic GmbH, Mannheim, Germany) and PhosSTOP (Cat. # 04906845001, Roche Diagnostic GmbH, Mannheim, Germany). Samples were centrifuged at 13,000 rpm for 20 min at 4 °C. The recovered supernatant was collected as the soluble protein fraction and again centrifuged to remove all insoluble protein and stored at −80°C [15]. The remaining pellets containing insoluble fractions were resuspended in the buffer RIPA and 8 M Urea. Samples were sonicated at 30–40% power for 7 s. The concentration of protein was determined using a BCA protein assay kit (Cat. # 23227: Pierce, Thermo Fisher Scientific, Rockford, IL, USA). A total of 10 µg protein was separated through a 10% gradient polyacrylamide gel and transferred to PVDF membrane (Cat# 1620177, Bio-Rab Laboratories, Inc, Hercules, CA, USA). 5% skim milk in 1× TBS-Tween 20 0.1 % (Tris-Buffered Saline -T) was used to block non-specific binding for 1 h and membranes were probed with primary antibodies at 4 °C overnight. The membranes were extensively washed with TBST and then incubated in horseradish peroxidase (HRP) conjugated secondary antibody for 1 h at room temperature. Finally, ECL reagent (Pierce, Thermo Fisher Scientific, Rockford, IL, USA) was used to detect protein and visualized using Image Quant LAS 500 (VWR, International, Radnor, PA, USA). Protein band density was quantified using Image Studio Lite Verison 5.2 (LI-COR, Lincoln, NE, USA).

### 2.9. Antibodies

RIPK3 (1:2000 cat# NBP1-77299, Novus Biologicals, Centennial, CO, USA), internal control GAPDH (1:100,000, Cat# MAB374, Sigma-Aldrich, St Louis, MO, USA), β-ACTIN (1:2000, Cat# 4967, Cell Signaling Technology, Inc., Danvers, MA, USA), RIPK1(1:1000, Cat# MAB3585, R&D systems, Inc., Minneapolis, MN, USA), HIF1α (1:1000, Cat# 3716, Cell Signaling Technology, Inc., Danvers, MA, USA), cleaved CASPASE3 (1:5000, Cat# 9664, Cell Signaling Technology, Inc., Danvers, MA, USA), MLKL (1:2000, Cat# MABC604, Millipore Corp, Burlington, MA, USA), CASPASE1 (1:100, Cat# sc-56036, Santa Cruz Biotechnology, Inc., Dallas, TX, USA) and CASPASE8 (1:100, Cat# sc-81656, Santa Cruz Biotechnology, Inc., Dallas, TX, USA) primary antibodies were used for the study. Horseradish peroxidase (HRP) conjugated goat anti-rabbit secondary antibody (Cat# 656120, Invitrogen, Thermo Fisher Scientific, Hanover Park, IL, USA) was used for cleaved CASPASE3 (1:100,000), RIPK3, HIF1α, CASPASE8, CASPASE1 (1:20,000), goat anti-rat secondary antibody (Cat# 629320, Invitrogen, Thermo Fisher Scientific, IL, USA) was used for MLKL (1:20000) and goat anti-mouse secondary antibody (Cat# G21040, Invitrogen, Thermo Fisher Scientific, IL, USA) was used for RIPK1 (1:20,000) and GAPDH (1:100,000).

### 2.10. Flow Cytometry

After 12 h of OGD, FFA treated AML-12 cells were washed with cold PBS twice and were trypsinized. The cells were resuspended in 500 µL of 1× binding buffer (Annexin V- FITC kit Cat# ab14085, Abcam, Cambridge, MA, USA), followed by addition of five µL of Annexin V-FITC and five µL of Propidium iodide (PI) and incubated for 15 min in the dark at room temperature. The samples were analysed using flow cytometry (Fortessa X-20, BD Biosciences, San Jose, CA, USA) and results were analysed using FlowJo (Version 10.6.1, Becton, Dickinson and Company, Franklin Lakes, NJ, USA).

### 2.11. Knockdown of MLKL and RIPK3 Expression Using Transient Transfection of MLKL siRNA and RIPK3 siRNA Respectively

MLKL was knocked down by RNA interference using MLKL siRNA (Cat# 4390771, ID: s92950 Silencer Select, Ambion, Life Technologies Corporation, New York, NY, USA). Similarly, RIPK3 was knocked down by RNA interference using RIPK3 siRNA (Cat# 4390771, ID: s80754, Silencer Select, Ambion, Life Technologies Corporation, New York, NY, USA). Silencer Negative control (Cat# 4390843, Silencer Select, Ambion, Life Technologies Corporation, New York, NY, USA) was used as negative control and GAPDH (Cat# 4390849, Silencer Select, Ambion, Life Technologies Corporation, New York, NY, USA) was used as positive control. Briefly, AML12 cells were seeded in a 12 well plate, 24 h prior to the transfection treatment. By following the manufacturer’s guidelines, siRNA was mixed with Lipofectamine RNAiMAX (Cat# 13778-150, Invitrogen, Life Technologies Corporation, NY, USA) in Opti-MEM I (Cat# 31985-070, Gibco, Life Technologies Corporation, NY, USA) and incubated for 15 min prior to transfer of the cells to 37 °C for 48 h. Then the transfected cells were prepared for FFA treatment followed by OGD exposure.

### 2.12. Enzyme-Linked Immunosorbent Assays and Lactate Dehydrogenase Assay

The concentration of tumour necrosis factor alpha (TNFα) and interlukin 6 (IL6) in conditioned media were measured using a mouse TNFα Enzyme-Linked Immunosorbent Assays (Elisa) kit (EK-0005, ElisaKit.com, Scoresby, VIC, Australia) and a mouse IL6 Elisa kit (Cat# EK-0029, ElisaKit.com, Scoresby, VIC, Australia) according to the manufacturer’s instructions. Similarly, LDH were determined with a lactate dehydrogenase (LDH) cytotoxicity assay kit (Cat# 601170, Cayman chemical, Michigan, MI, USA) according to the manufacturer’s instructions.

### 2.13. Statistical Analysis

All data obtained were presented as mean ± standard deviation (SD) and Student’s t-test was used to compare the statistical significance between two groups. For statistical analysis and graphical presentation of results, GraphPad Prism software (Version 8, GraphPad Software, San Diego, CA, USA) was used. For all tests, a *p* < 0.05 was accepted as statistically significant.

## 3. Results

### 3.1. Development of an In Vitro Model of Fatty Liver Undergoing Ischaemic Injury

#### 3.1.1. Optimization of FFA Treatment in AML-12 Cells

**Figure 1:**
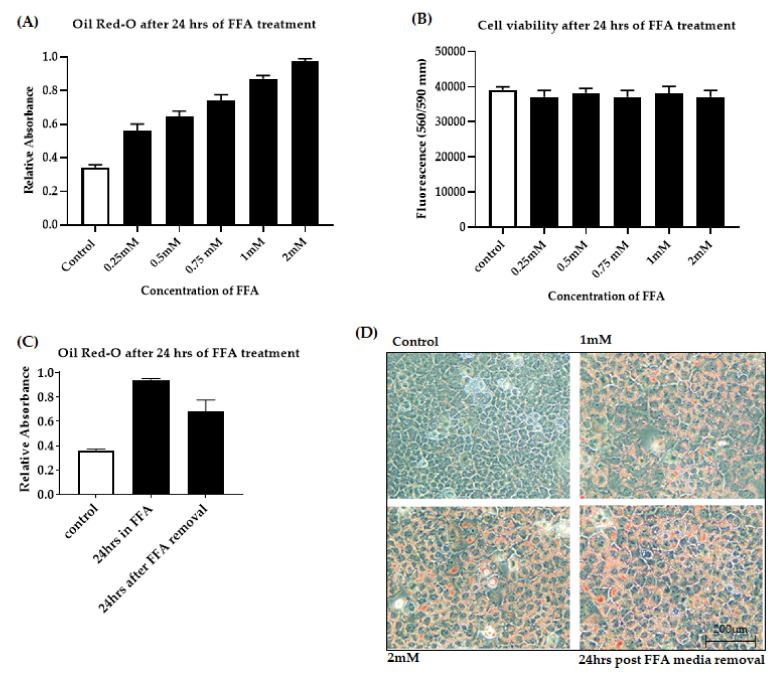
Free fatty acid accumulation in AML-12 cells. Cells were exposed to increasing concentrations of FFA from 0 to 2 mM. (**A**): Dose-dependent FFA accumulation was quantified by measuring the absorbance of the lipophilic dye Oil-red O. (**B**): Cell viability was assessed by fluorometric quantitation. (**C**): Lipid accumulation was quantified by measuring the absorbance of oil-red O after 24 h FFA removal. (**D**): Intracellular fat accumulation measured by Oil-red O staining at 20× magnification. Data is represented as mean ± SD from 3 independent experiments. alpha mouse liver 12 (AML-12) cell line, Free fatty acid (FFA).

Primary human hepatocytes represent the “gold standard” for studying metabolic regulation at the cellular level. However, due to their limited availability and variability in quality between donors, we used the murine immortalized cell line AML-12. We favored the use of AML-12 hepatocytes because they were originally derived from healthy liver cells. In addition, they exemplify normal fatty acid metabolism that closely resembles that of primary murine hepatocytes [25], allowing a direct transposition of the results obtained in mice. In our study, AML-12 cells were treated with a combination of sodium salts of oleate and palmitate during FFA treatment. Both oleic (C18:1) and palmitic (C16:0) acids are the most abundant fatty acids found in the steatotic liver [26]. A growing body of literature demonstrates the successful use of these fatty acids for steatosis induction in a mouse model [27], immortalized hepatocyte cell lines [28,29] and primary mouse hepatocyte culture [29,30]. In this study, we have used a 2:1 ratio of sodium salts of oleate and palmitate as this ratio shows lower cytotoxic effects even in higher concentration [31]. A dose-dependent increase in fat accumulation was observed after 24 h of FFA treatment. To confirm fat accumulation in hepatocytes, microscopic analysis was performed after oil-red O staining. The microscopic findings were then verified by absorbance spectrophotometry, which showed dose-dependent intracellular fat accumulation after 24 h of exposure (Figure 1A). There was no significant decrease in cell viability after FFA exposure (Figure 1B). 2 mM FFA was considered to be optimal for OGD treatment as the cells maintained viability and FFA deposition even after 24 h of FFA media removal as shown in Figure 1C,D.

#### 3.1.2. OGD Treatment Decreases Cell Viability in an In Vitro Model of Steatosis

**Figure 2:**
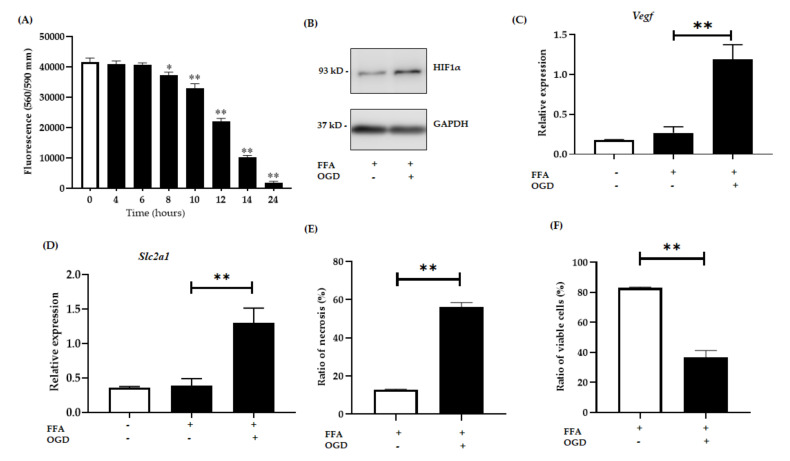
Relative expression of hypoxia sensitive genes. AML-12 cells were treated with 2 mM FFA for 24 h and then subjected to 12 h of OGD. (**A**): Cell viability was assessed by fluorometric quantitation at 0, 6, 8, 10, 12, 14 and 24 h of OGD. (**B**): Relative protein expression in FFA control cells and FFA + OGD cells were detected by western blot analysis; upper panel HIF1α and lower panel GAPDH. (**C**): Relative mRNA expression of *Slc2a1*. (**D**): Relative mRNA expression of *Vegf*. (**E**,**F**): Cells were stained with annexin fluorescein and propidium iodide and analysed by flow cytometry for the ratio of necrotic cells and percentage of viable cells respectively. Data is represented as mean ± SD from 3 independent experiments. Comparison between OGD treated groups and the untreated control at * *p* < 0.05, ** *p*< 0.0001. Alpha mouse liver 12 (AML-12) cell line, Free fatty acid (FFA), oxygen glucose deprivation (OGD), Hypoxia-inducible factor-1 alpha (Hif1α), Glyceraldehyde 3-phosphate dehydrogenase (GAPDH), messenger RNA, solute carrier family 2, facilitated glucose transporter member 1 (*Slc2a1*), vascular endothelial growth factor (*Vegf*).

The OGD model has been frequently used in the study of I/R injury in vitro. In the OGD model, cells were grown in normal culture conditions replete with glucose and oxygen and then moved into an environment lacking both glucose and oxygen for a time-course to mimic ischaemic injury [32,33]. The successful use of the OGD model to mimic the pathogenesis of I/R insult is well described in the literature, enabling the elucidation of the underlying mechanisms of ischaemic injury [33,34]. To confirm the most optimal OGD time for FFA treated AML-12 cells, we exposed the FFA treated cells to OGD condition at various time points (4 h, 6 h, 8 h, 10 h, 14 h and 24 h). Cell viability assay revealed that the viability of cells exposed to 4 h and 6 h of OGD were not significantly decreased compared to cells grown in normal conditions (Figure 2A). Whereas, cells exposed to 8 h (*p* < 0.05), 10 h, 12 h, 14 h and 24 h showed significant loss of cell viability (all *p* < 0.0001) (Figure 2A). Cells exposed to 14 h and 24 h of OGD, suffered dramatically and resulted in cell viabilities of 24.59 ± 1.39% and 4.42 ± 1.32% respectively. Considering the above results, we decided the OGD exposure of 12 h was optimal for our study with 47.15 ± 1.60% loss of cell viability compared to controls. For the reoxygenation phase, after 12 h of OGD exposure, the injured cells were placed back into normal growth media and supplied with adequate oxygen for various time points (1, 3, 6, 9 h) to mimic the reperfusion phase. However, reoxygenation after OGD exposure failed to rescue the cells from cell death (Figure S1) and after 9 h of reoxygenation, viability of cells was further compromised. Considering above results, we decided to focus on ischemia and not on the reperfusion stage in this study.

Hypoxia-inducible factor-1 alpha (*Hif1α*) which activates in hypoxic conditions was used as an indicator for hypoxia in our experiment [35,36]. After 12 h of OGD exposure, a significant increase in the expression of HIF1α was observed in FFA + OGD treated cells compared to FFA control cells (Figure 2B). Numerous studies have shown activation of Nuclear factor kappa light chain enhancer of activated B cells (*NF-kB*) upon hypoxia [35,36] which in turn activates target genes such as *Vegf* [37,38]. Several studies have shown the activation of *Vegf* upon ischaemia or under hypoxic condition [39,40,41]. Interestingly, activation of Hif1α also triggers *Slc2a1* activation [42]. Hence to further validate the hypoxia induction and activation of *Hif1α*, we examined mRNA expression of *Vegf* and *Slc2a1* in FFA + OGD treated cells. FFA + OGD treated cells showed increased expression of both *Slc2a1* (*p* < 0.0001) and *Vegf* (*p* < 0.0001) compared to FFA treated cells grown in normal conditions (Figure 2C,D). Flow cytometry assay revealed that OGD exposure increased the percentage of necrotic cells compared to control FFA cells (Figure 2E). Further, loss of viability was also verified by flow cytometry in FFA + OGD treated cells (*p* < 0.0001) (Figure 2F).

### 3.2. OGD Treatment Elevated the Insoluble Fraction of RIPK1, RIPK3 and MLKL Protein in FFA Treated AML-12 Cells

**Figure 3:**
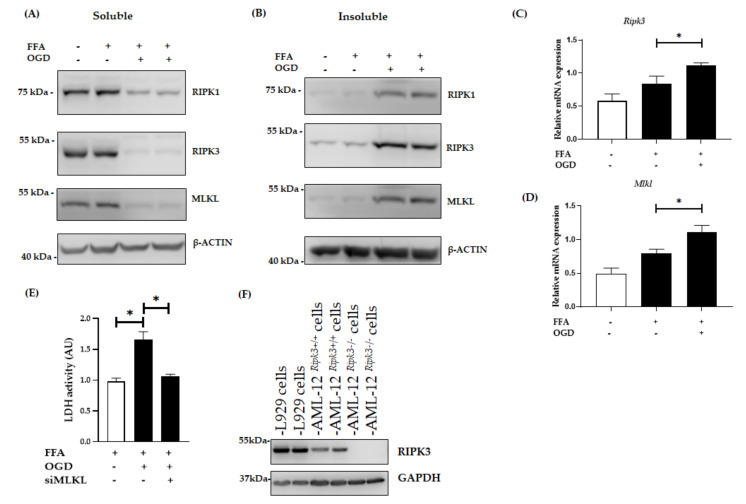
Expression of necroptotic proteins in FFA + OGD treated AML-12 cells. Relative protein expression in FFA control cells and FFA + OGD cells were detected by western blot analysis; using antibodies against RIPK1, RIPK3 and MLKL. β-ACTIN was used as the loading control. (**A**): Soluble protein (**B**): Insoluble protein. (**C**): Relative *Ripk3* mRNA expression in FFA control cells and FFA + OGD cells. (**D**): Relative *Mlkl* mRNA expression in FFA control cells and FFA + OGD cells. (**E**): Cellular necrosis was measured as LDH release after 12 h of OGD treatment. *(***F**): Validation of RIPK3 antibodies and expression of RIPK3 in AML-12 cells. Data is represented as mean ± SD from 3 independent experiments. Comparison of cell viability between siMLKL transfected FFA + OGD treated groups and non-transfected FFA + OGD at * *p* < 0.05. alpha mouse liver 12 (AML-12) cell line, Free fatty acid (FFA), oxygen glucose deprivation (OGD), receptor-interacting protein kinase 1 (RIPK1), receptor-interacting protein kinase 3 (RIPK3), mixed-lineage kinase domain-like pseudokinase (MLKL), Beta-actin (β-ACTIN), lactate dehydrogenase (LDH).

RIPK1 is known to be an initiator of the necroptosis pathway and RIPK3 has emerged as a central player of necroptosis whereas MLKL is known to be the key mediator in necroptotic cell death working downstream of RIPK3 [21,43,44]. Thus, to investigate whether OGD treatment amplifies RIPK1, RIPK3 and MLKL expression in our in vitro model of hepatic ischaemia, we explored their protein level using western blot analysis. Interestingly, after the exposure of FFA treated cells to OGD conditions, RIPK1, RIPK3 and MLKL expression significantly sequestered in an insoluble fraction compared to control FFA treated cells (Figure 3A,B) indicating activation of the RIPK1-RIPK3-MLKL dependent pathway. The formation of the amyloid-like structure of the necrosome is considered to be a critical step during pro-necroptotic signaling [15,45]. The retention of these proteins in an insoluble fraction in our model supports this phenomenon. Further, PCR results showed that the mRNA expression of RIPK3 and MLKL were higher in FFA + OGD treated cells compared to the FFA controls (*p* < 0.05) (Figure 3C,D). We then analysed lactate dehydrogenase (LDH) release, which is considered a characteristic feature seen in necrotic cell death [46,47]. We demonstrated that the release of LDH was significantly increased in FFA + OGD treated cells compared to the FFA controls (*p* < 0.05) (Figure 3E). We also analysed the effect of OGD on untreated AML-12 cells and we found that both RIPK3 and MLKL were sequestered in the insoluble fraction upon OGD exposure as seen during FFA + OGD treatment (Figure S2).

There has been controversy surrounding the expression of RIPK3 in hepatocytes as well as the efficacy of commercially available RIPK3 antibodies. A previous study has shown the expression of RIPK3 in AML-12 cells [48]. Hence, to address this issue we used CRISPR-generated AML-12*^Ripk3−/−^* cells lines and L929 cells, which are known to over-express RIPK3, as a positive control for RIPK3 expression. Western blot analysis revealed that RIPK3 expression in control AML-12 cells was lower than L929 cells and there was an absence of RIPK3 in AML12*^Ripk3−/−^* cells (Figure 3F).

### 3.3. Inhibition of MLKL, and RIPK3 Attenuated the Necroptotic Cell Death Induced by OGD

**Figure 4:**
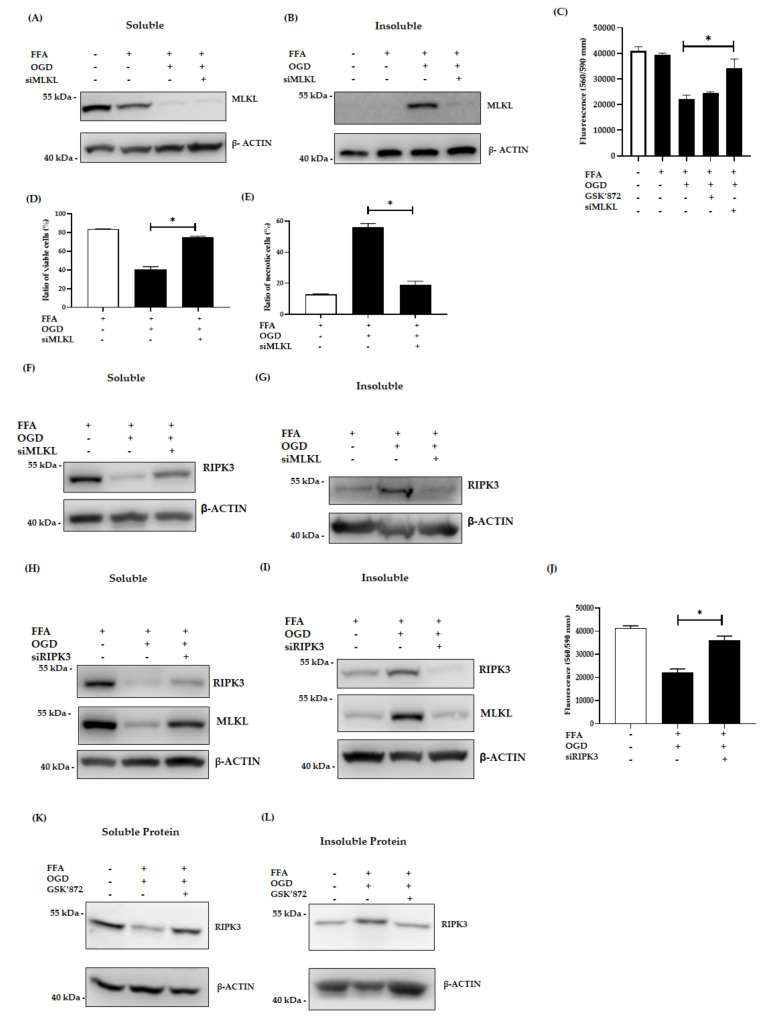
Effect of siMLKL, siRIPK3 and GSK’872 treatment in FFA + OGD exposed AML-12 cells. (**A**,**B**): Soluble and insoluble MLKL protein expression after siMLKL treatment post OGD in FFA treated AML-12 cells respectively. (**C**): Cell viability was assessed by fluorometric quantitation. (**D**,**E**): Cells were stained with Annexin fluorescein and propidium iodide and analysed by flow cytometry for the ratio of percentage of viable cells and necrotic cells respectively. (**F**,**G**): soluble and insoluble RIPK3 protein expression siMLKL transfect FFA + OGD treated AML-12 cells respectively. (**H**,**I**): Soluble and insoluble RIPK3 and MLKL protein expression after siRIPK3 treatment post OGD in FFA treated cells respectively. (**J**) Cell viability was assessed by fluorometric quantitation. (**K**,**L**): Soluble and insoluble RIPK3 protein expression after 50 µm of GSK’872 treatment post OGD in FFA treated AML-12 cells respectively. β-ACTIN was used as the loading control for western blot. Data is represented as mean ± SD from 3 independent experiments. Comparison of cell viability in respective siMLKL and siRIPK3 transfected FFA + OGD treated groups and non-transfected FFA + OGD at * *p* < 0.0001. small interfering (si), mixed-lineage kinase domain-like pseudokinase (MLKL), receptor-interacting protein kinase 3 (RIPK3), Free fatty acid (FFA), oxygen glucose deprivation (OGD), alpha mouse liver 12 (AML-12) cell line, Beta-actin (β-ACTIN).

Previous studies have demonstrated the crucial role of RIPK3 and MLKL in activation of the necrosome and in the necroptotic signalling pathways [14,15,45]. Thus we examined the relative expression of RIPK3 and MLKL in our model by applying different intervention techniques. For this, AML-12 cells were transfected with siMLKL to knockdown the expression of MLKL. Then the cells were treated with FFA for 24h followed by OGD exposure. Intervention with MLKL siRNA inhibited the relative expression of both soluble and insoluble MLKL as shown by western blot (Figure 4A,B). Further, the siMLKL transfected cells showed decreased expression of MLKL even after OGD exposure. Interestingly, siMLKL intervention increased viability of OGD treated cells compared to the OGD non-transfected control cells as demonstrated by cell viability assay (*p* < 0.0001) (Figure 4C). This result is further confirmed by flow cytometry where the siMLKL treated cells showed a significant increase in the percentage of viable cells and reduction in the ratio of necrotic cells even after exposure to FFA + OGD (Figure 4D,E). Additionally, LDH release was decreased in siMLKL treated FFA + OGD exposed cells (Figure 3E). Then we also looked into the effect of siMLKL treatment on the expression of RIPK3 in FFA + OGD cells. Intriguingly, the soluble fraction of RIPK3 increased and insoluble fraction decreased in siMLKL treated FFA + OGD cells which was reversed in FFA + OGD non transfected cells (*p* < 0.0001) (Figure 4F,G). To evaluate the involvement of RIPK3 in our model in addition to the expression of the insoluble fraction, we used siRIPK3 to knockdown RIPK3 expression in FFA + OGD cells. Our results revealed that RIPK3 siRNA decreased the expression of RIPK3 in FFA + OGD treated cells (Figure 4H,I). siRIPK3 intervention was also able to improve the viability of FFA + OGD cells compared to non-transfected FFA + OGD control cells (*p* < 0.0001) (Figure 4J). We also confirm that the siRIPK3 treatment increased the soluble fraction of MLKL and decreased the insoluble fraction in FFA + OGD cells (Figure 4H,I).

To investigate the therapeutic potential of RIPK3 inhibitor in our model, we used GSK’872, a RIPK3 kinase inhibitor and explored its effect in our model. Pre-treatment with the inhibitor prior to OGD was more effective than post-OGD treatment. In western blot analysis, insoluble retention of RIPK3 expression was decreased after 50 µm GSK’872 pre-treatment (Figure 4K,L). However, cell viability assay revealed that GSK’872 treatment failed to rescue the cells from OGD insults (Figure 4C). GSK’872 is cytotoxic at higher concentrations and cells can undergo caspase dependent apoptosis as described by other studies [33,49,50]. In our study, we treated the cells with 50µm GSK’872 which showed the presence of cleaved-CASPASE3, indicating a potential switch of cell death to apoptosis (Figure S3).

### 3.4. Changes in Pro-Inflammatory Cytokine Secretion after FFA and OGD Treatment in AML-12 Cells

**Figure 5:**
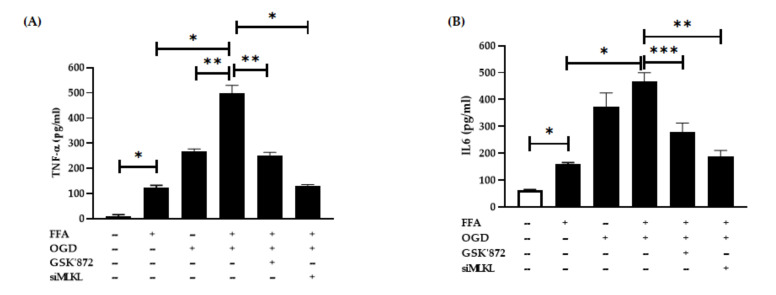
Proinflammatory cytokines expression after OGD treatment. (**A**) TNFα (**B**) IL6. Comparison between OGD treated groups and the untreated control at * *p* < 0.0001, ** *p* < 0.001 and *** *p* < 0.01. Free fatty acid (FFA), Oxygen glucose deprivation (OGD), tumour necrosis factor alpha (TNFα), interlukin-6 (IL6).

Certain pro-inflammatory cytokines such as TNFα and IL6 have been linked with ischaemic injury. Hence, we investigated their post-OGD secretion in conditioned media using ELISA assays. Consistent with other studies, we found that FFA treatment alone markedly elevated the TNFα and IL6 secretion in FFA treated cells compared to the controls (both *p* < 0.0001) (Figure 5A,B). Further, FFA + OGD exposed cells showed drastic increase in the secretion of both TNFα and IL6 when compared to FFA treated cells (both *p* < 0.0001). Moreover, TNFα expression was higher when compared with OGD alone indicating that combined treatment of FFA + OGD further exacerbated the cell injury. However, FFA + OGD-induced secretion of TNFα level was significantly suppressed in cells treated with GSK’872 (*p* < 0.001) and even greater suppression in siMLKL treated cells (*p* < 0.0001). Similarly, individual treatment with GSK’872 and siMLKL significantly reduced the secretion of IL6 (*p* < 0.01 and *p* < 0.001 respectively) in FFA + OGD cells. These results indicated that inhibition of RIPK3 and MLKL may reduce cellular injury and production of pro-inflammatory cytokines in FFA + OGD treated AML-12 cells.

### 3.5. Apoptosis, Pyroptosis and Ferroptosis Were Not Active Pathways in FFA + OGD Exposed AML-12 Cells

**Figure 6:**
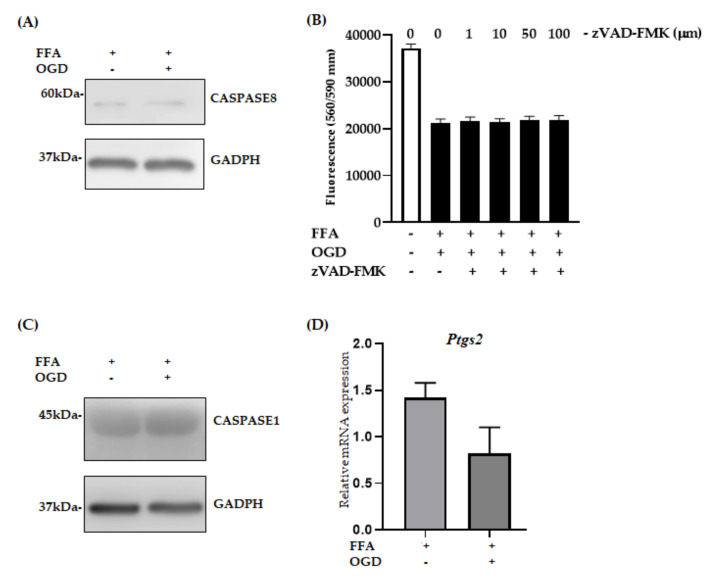
Relative expression of apoptosis, pyroptotsis and ferroptosis markers in FFA + OGD cells. (**A**) Relative protein expression in FFA control cells and FFA + OGD cells were detected by Western blot analysis; upper panel CASPASE8 and lower panel GAPDH. GAPDH was used as the loading control. Western blot results were quantitated using Image Studio Lite Verison 5.2 (LI-COR, Lincoln, Nebraska USA). (**B**) Cell viability of z-VAD-fmk treated FFA + OGD exposure cells was assessed by fluorometric quantitation. (**C**) Relative protein expression in FFA control cells and FFA + OGD cells were detected by Western blot analysis; upper panel CASPASE1 and lower panel GAPDH. (**D**) Relative *Ptgs2* mRNA expression in FFA control cells and FFA + OGD cells. Free fatty acid (FFA), oxygen glucose deprivation (OGD), Glyceraldehyde 3-phosphate dehydrogenase (GAPDH), prostaglandin-endoperoxide synthase 2 (Ptgs2), messenger RNA (mRNA).

To investigate the possibility that other cell death pathways are activated following OGD in AML-12 cells, we performed a series of analyses. The activation of CASPASE is the hallmark feature of apoptosis [51]. Hence, to investigate if apoptosis is active in our in vitro model, we evaluated the protein expression of CASPASE8 and cleaved-CASPASE3. Upon OGD exposure, no significant activation of CASPASE8 expression was seen (Figure 6A). In addition, the absence of cleaved-CASPASE3 (Figure S3) strongly indicated that apoptosis was not an active pathway in this model. Further, we pre-treated the FFA loaded AML-12 cells with different concentrations of zVAD-FMK, a known inhibitor of apoptosis to analyse if it can halt the cell death process after OGD exposure. Viability of FFA + OGD treated cells with zVAD-FMK (1–100 µm) did not improve compared to FFA + OGD cells (Figure 6B). Similarly, we performed a preliminary investigation of other cell death pathways in our model. We did not observe any difference in expression of CASPASE1, a key marker for pyroptosis in FFA + OGD treated cells compared to FFA controls (Figure 6C). Additionally, ferroptosis, another form of cell death, is known to involved in hepatic I/R injury [24]. Hence, we examined the mRNA expression of *Ptgs2*, a marker of ferroptosis in our FFA + OGD treated cells. We found that there was no significant difference in the expression pattern of *Ptgs2* in FFA + OGD compared to FFA control cells (Figure 6D). These results suggested that other cell death mechanisms are unlikely to be involved in our in vitro model.

## 4. Discussion

During liver transplantation, the donor liver is likely to encounter I/R injury which involves various molecular and biological mechanisms leading to cell death. Ischaemic injury can trigger multiple molecular mechanisms such as necroptosis, a novel form of cell death which has been recently observed in steatotic hepatic I/R injury [16,17]. In this study, we developed an in vitro model of hepatic steatosis undergoing ischaemic injury and investigated the underlying mechanism of cell death. We used an in vitro murine hepatocyte cell line for our study since there has been increasing evidence to support the use of in vitro models to investigate the effect of steatosis in hepatocytes. Likewise, the OGD model has been proven as a suitable tool to study ischaemic effects [33]. In our study, we confirm the existence of necroptosis in our in vitro hepatocyte steatosis and hypoxia model as evidenced by alterations in the expression of RIPK1, RIPK3 and MLKL. Additionally, preliminary exploration of other cell death pathways in our model suggested they were not operative.

Emerging studies have demonstrated the critical involvement of RIPK3 in I/R injury in various organ systems including the liver. RIPK3 overexpression was seen in an in vitro model of hippocampal neuronal HT-22 cells undergoing OGD injury [33]. Likewise, similar findings were observed in hypoxic-ischaemic retinal disease studies using retinal ganglion cell 5 (RGC-5) cells [52]. RIPK3 executes necroptosis via phosphorylation of MLKL that translocates to the cell membrane for its subsequent lysis. Thus MLKL is considered a key player in necroptosis [21]. In our study, we found that RIPK1, RIPK3 and MLKL were significantly sequestered in an insoluble fraction of the protein providing strong evidence of necrosome formation. This insoluble form of these proteins and formation of the amyloid-like necrosome has been previously documented [15,45]. Further, LDH leakage is a hallmark feature of necrotic cell death [46,47]. In a previous study, LDH release and activation of RIPK3 and MLKL were seen in drug induced cell death [47]. In the present study, we observed increased release of LDH from FFA + OGD exposed cells compared to the FFA controls. Additionally, we also showed an increase in necrotic cells upon ischaemic insults. These finding provide evidence that AML-12 cells in our model have undergone necrotic cell death.

It is evident that the formation of the necrosome through the binding of RIPK1, RIPK3 and MLKL is essential for the induction of necroptosis. In our study, we have shown that siMLKL treatment downregulated the expression of MLKL in OGD exposed FFA treated cells. Interestingly, siMLKL treatment decreased the insoluble fraction of RIPK3 and increased the soluble fraction in FFA + OGD treated cells. In our study, MLKL siRNA significantly attenuated the cell injury, reduced the necrotic form of cell death and ameliorated cell death post OGD exposure. Further, we report that siRIPK3 treatment was able to protect the cells from ischaemic insult. We have also shown that siRIPK3 treatment reduced the insoluble MLKL fraction and increased in soluble form. Considering these siRNA intervention results, both RIPK3 and MLKL may have failed to create amyloid like necrosome in the absence of one another and hence the assembly of necrosomal components was possibly halted. This finding provides further evidence for the involvement of RIPK3 and MLKL in OGD induced cell death in our model and suggest that necroptosis is operated via RIPK3-MLKL axis in our in vitro model. We also propose that both RIPK3 and MLKL perhaps require RIPK3-MLKL axis to form insoluble necrosome since both of them failed to go into insoluble fraction when the other one was knocked down. Additional experiments are indeed required and possible use of recently developed phospho-antibodies will help to decipher the underlying molecular mechanisms.

Studies have shown that the inhibition of RIPK3 ameliorates cell death in many different organ systems and is used as potential therapeutic target [33,53]. To investigate the therapeutic potential of RIPK3 inhibitor in our model, we used GSK’872 a RIPK3-selective kinase inhibitor and which has been effectively used as a potential therapeutic target of RIPK3 activity during necroptosis. Here, we found that pre-treatment with GSK’872 reduced the expression of insoluble RIPK3 in FFA + OGD treated cells. Contrasting with the above-mentioned studies, GSK’872 treatment was unable to protect the cells from OGD insult as cell viability did not improve. The potential reason behind this outcome is that GSK’872 is cytotoxic at higher concentration as described by other studies [33,49,50]. Previously, when necrosis sensitive murine L929 cells were exposed to pro-necrotic mutant virus, the presence of 10µm GSK’872 drastically decreased the cell viability which was reversed by additional zVAD treatment [50]. This data has shown the caspase dependent cytotoxicity of GSK’872 at higher dose. Another study has also demonstrated the spontaneous toxicity of GSK’872 at higher doses leading to apoptosis [49]. In our study, we used a higher concentration of GSK’872 as lower concentrations did not reduce the expression of RIPK3 (Figure S3). It is undeniable that GSK’872 treatment could potentially switch cell death to apoptosis as we noticed the presence of cleaved-CASPASE3 after 50 µM GSK’872 treatment. Moreover, Most RIPK3 kinase inhibitors induce apoptosis in a concentration-dependent manner [54,55]. Hence, unfortunately RIPK3 cannot be pharmacologically targetable without side effects [54]. Use of newly developed RIPK3 inhibitor could potentially help answer the question as to whether RIPK3 can be pharmacologically targeted to minimize the cell injury in an ischaemic setting [54].

For a long period of time, apoptosis was the most widely understood mode of regulated cell death as it plays a critical role during tissue development and morphogenesis of multicellular eukaryotes [51,56]. On the other hand, necroptosis has been identified as a novel form of programmed cell death that involves many proteins that been suggested to play a role as instigators, executors and effectors of necroptosis [57,58,59,60,61]. Only recently, have key molecular mechanisms of necroptosis been established. At the cellular level, necroptosis is characterized by cytoplasmic granule formation, organelle swelling leading to loss of membrane permeability and disintegration of the plasma membrane [62,63,64]. This is followed by release of intracellular organelles and damage- associated molecular pattern molecules (DAMPs), sending inflammatory signals to adjacent cells [61,65,66]. Overexpression of inflammatory cytokines such as TNFα and IL6 in steatotic liver has been previously well-documented, as has increased production of TNFα and IL6 in IR injury [67]. Our study has also shown the increased expression of both TNFα and IL6 after FFA treatment and significantly higher expression of TNFα and IL6 in FFA + OGD treated cells compared to FFA cells. Though we found the expression patterns of RIPK3 and MLKL were similar in FFA + OGD and OGD alone groups, both TNFα and IL6 were greater in FFA + OGD groups compared to the OGD alone indicating that the presence of FFA exacerbated the injury in FFA + OGD when compared to OGD alone. Our study also demonstrated that siMLKL and GSK’872 treatment decreased the secretion of TNFα and IL6 indicating that targeting of RIPK3 and MLKL can attenuate cellular injury during ischaemic injury in OGD treated cells.

Numerous studies have shown the activation of necroptosis through various receptors and the TNFα mediated pathway of necroptosis has been widely studied. Upon activation of RIPK3, if CASPASE8 is activated it can trigger the caspase cascade signaling, resulting in cleavage of CASPASE3 and then the cell will undergo apoptosis [68]. However, if CASPASE8 is inhibited RIPK3 will recruit MLKL which result in rupturing of the plasma membrane and cell death [18,45]. There has been controversy surrounding which particular cell death pathway actively participates in hepatic IR injury. Some studies have shown the involvement of apoptosis during hepatic IR injury with TUNEL staining and caspase activity assay. However, recent investigations have shown convincing evidence of necroptosis without caspase activation during hepatic IR injury [16,17]. In our study, we did not observe a difference in the expression level of CASPASE8 and an absence of cleaved-CASPASE3 which provides further evidence that apoptosis did not participate in our model. Additionally, dose dependent zVAD-FMK exposure also did not improve the cell viability which further indicates that our model is independent of apoptosis. Apart from necroptosis and apoptosis, recent evidence demonstrates the emergence of new cell death pathways such as pyroptosis, ferroptosis and PAN-ptosis. Interestingly, in our study, there was no change in the expression level of CASPASE1 (marker for pyroptosis) suggesting that FFA + OGD cells did not undergo pyroptosis. Similarly, there was no significant difference in the mRNA expression of *Ptgs2*, a marker for ferroptosis, which indicates that ferroptosis may not have been involved in our model. Additional studies are indeed required to validate the non- involvement of pyroptosis and ferroptosis during hepatic steatosis with ischaemic injury.

## 5. Conclusions

Though necroptosis has been directly linked to I/R injury in other organ transplants, its role in fatty liver undergoing I/R injury has not been fully examined. In this project we hypothesized that hepatic necroptosis mediates cell death during ischaemic injury in fatty liver. Our hepatic steatotsis and ischaemia in vitro injury model suggested that necroptosis played an instrumental role in this model. Collectively, our data highlight that necroptosis is involved in ischaemic insults in FFA treated hepatocytes under OGD conditions by activating RIPK1, RIPK3 and MLKL. In addition, our results suggest that apoptosis, pyroptosis and ferroptosis are not activated in our in vitro model. Further, siMLKL and siRIPK3 protect the FFA treated cells against OGD-induced injury respectively. Both MLKL and RIPK3 may be promising pharmacological targets to inhibit necroptosis during I/R injury in fatty liver and allow expansion of the donor pool in liver transplantation. In summary, our findings provide a novel prospective for studies of necroptosis in fatty liver and ischaemic injury and suggest necroptosis is a potential therapeutic target.

## Figures and Tables

**Table 1:** Real time PCR primer sets. Pre-validated primer sequences from Origene (Rockville, MD, USA).

Gene	Primer	Sequence (5′ to 3′)
*Slc2a1*	Forward	GCTTCTCCAACTGGACCTCAAAC
	Reverse	ACGAGGAGCACCGTGAAGATGA
*Vegf*	Forward	CTGCTGTAACGATGAAGCCCTG
	Reverse	GCTGTAGGAAGCTCATCTCTCC
*Rpl27*	Forward	GCGATCCAAGATCAAGTCCTTTG
	Reverse	TCAAAGCTGGGTCCCTGAACAC
*RER1*	Forward	GACACTGGGCCTGAGTTTTG
	Reverse	GGAGAAAGGAACGCAATGAA
*18S*	Forward	AGTTGGTGGAGCGATTTGTC
	Reverse	AACGCCACTTGTCCCTCTAA
*Ptgs2* [24] *Ripk3* *Mlkl*	Forward	GGG AGT CTG GAA CAT TGT GAA
	Reverse Forward Reverse Forward Reverse	GTG CAC ATT GTA AGT AGG TGG ACT GAA GAC ACG GCA CTC CTT GGT A CTT GAG GCA GTA GTT CTT GGT GG CTGAGGGAACTGCTGGATAGAG CGAGGAAACTGGAGCTGCTGAT

## Data Availability

The data presented in this study are available on request from the corresponding author.

## Supplementary Materials

The following are available online at https://www.mdpi.com/2077-0383/10/2/212/s1. Figure S1. (A) Reoxygenation of AML-12 cells after 12 h of OGD exposure. Cell viability was assessed by fluorometric quantitation at 1, 3, 6 and 9 h of reoxygenation. Figure S2. Expression of necroptotic proteins in OGD exposed cells. Relative protein expression in control cells and OGD cells were detected by western blot analysis; using antibodies against RIPK3 and MLKL. β-ACTIN was used as the loading control. (A) Soluble protein (B) Insoluble protein. Β-ACTIN was used as the loading control. Figure S3. GSK’872 treatment of AML12 cells after FFA + OGD treatment. (A) Relative protein expression of cleaved+CASPASE3 in FFA + OGD cells and FFA + OGD + GSK’872 treated cells were detected by western blot analysis; upper panel cleaved-CASPASE3, lower panel GAPDH. GAPDH was used as the loading control. Western blot results were quantitated using Image 4.0 software.
