# Supplementary material for: Inhibition of MLKL Attenuates Necroptotic Cell Death in a Murine Cell Model of Hepatic Ischaemia Injury
Source: J Clin Med. 2021 Jan 8;10(2):212. doi: 10.3390/jcm10020212 (PMC7826539; doi:10.3390/jcm10020212)
Supplement: Supplementary file 1 [file jcm-10-00212-s001.pdf]

Journal of
*Clinical Medicine*

MDPI

*Supplementary material*

# Inhibition of MLKL attenuates necroptotic cell death in a murine cell model of ischaemia injury

**Raji Baidya [1,2], Jérémie Gautheron [3,4], Darrell H. G. Crawford [1,2], Haolu Wang [2,5] and Kim R. Bridle [1,2],***

1 Faculty of Medicine, The University of Queensland, Brisbane, 4006, Queensland, Australia; r.baidya@uqconnect.edu.au (R.B.); d.crawford@uq.edu.au (D.H.G.C.)
2 Gallipoli Medical Research Institute, Brisbane, 4120, Queensland, Australia; h.wang21@uq.edu.au
3 Sorbonne University, Inserm, Centre de Recherche Saint-Antoine (CRSA), Paris, 75012, France; jeremie.gautheron@inserm.fr
4 Institute of Cardiometabolism and Nutrition (ICAN), Paris, 75013, France
5 Diamantina Institute, The University of Queensland, Brisbane, 4102, Queensland, Australia
* Correspondence: k.bridle@uq.edu.au; Tel.: +61-7-3346-0698

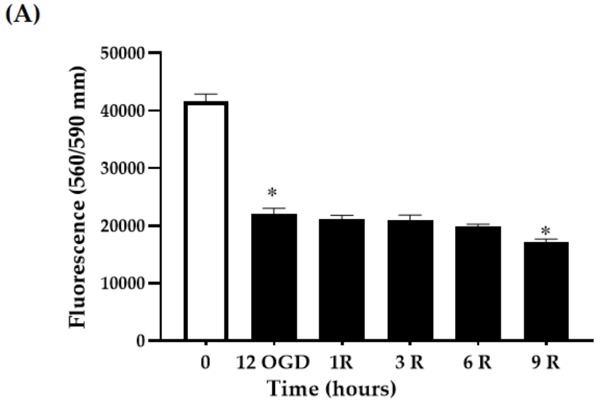


**Figure S1.** (A) Reoxygenation of AML-12 cells after 12 h of OGD exposure. Cell viability was assessed by fluorometric quantitation at 1, 3, 6 and 9 h of reoxygenation. *p< 0.0001.

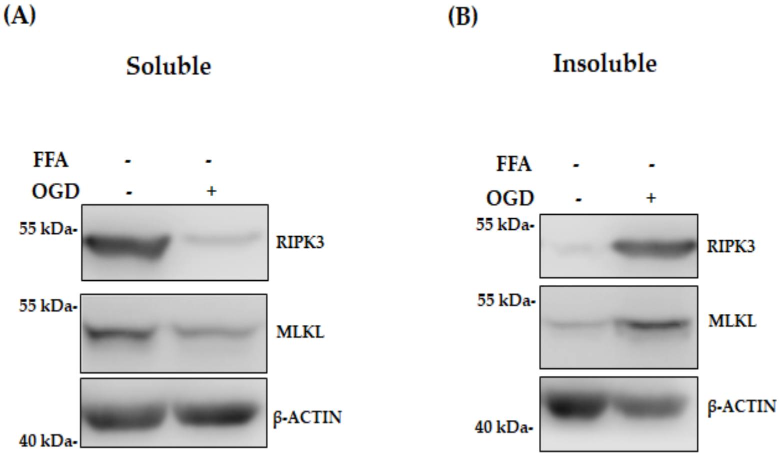


**Figure S2.** Expression of necroptotic proteins in OGD exposed cells. Relative protein expression in control cells and OGD cells were detected by western blot analysis; using antibodies against RIPK3 and MLKL. β-ACTIN was used as the loading control. A) Soluble protein B) Insoluble protein. B-ACTIN was used as the loading control.

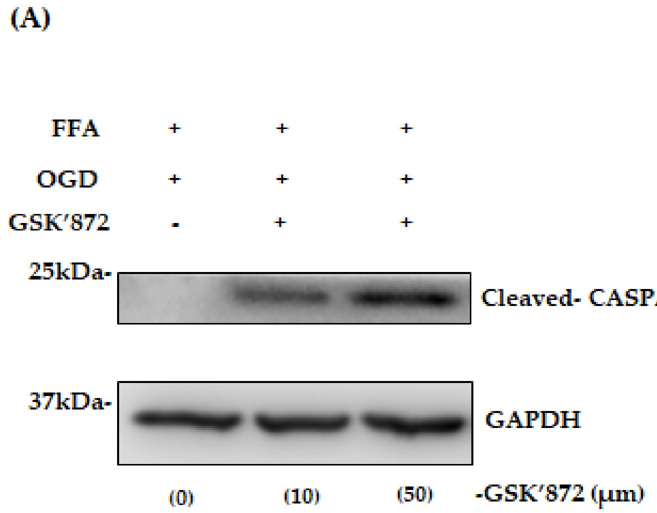


**Figure S3.** GSK'872 treatment of AML12 cells after FFA+OGD treatment. A) Relative protein expression of cleaved+CASPASE3 in FFA+OGD cells and FFA+OGD+GSK'872 treated cells were detected by western blot analysis; upper panel cleaved-CASPASE3, lower panel GAPDH. GAPDH was used as the loading control. Western blot results were quantitated using Image Studio Lite Verison 5.2.
